# Intention to disclose medical errors by healthcare providers to patients and associated factors in Ugandan health facilities: A cross-sectional study

**DOI:** 10.1371/journal.pgph.0005973

**Published:** 2026-02-09

**Authors:** Catherine Alupo, Jonathan Izudi, Christopher Ddamulira

**Affiliations:** 1 School of Public Health, Bugema University, Kampala, Uganda; 2 Department of Community Health, Mbarara University of Science and Technology, Mbarara, Uganda; 3 Directorate of Graduate Training, Research and Innovation, Muni University, Arua, Uganda; School of Public Health, College of Health Science, Addis Ababa University, ETHIOPIA

## Abstract

This study assessed the prevalence and factors associated with intention to disclose medical errors by healthcare providers to patients in public health facilities in Mukono District, Uganda. We conducted an analytic cross-sectional study among healthcare providers across four public health facilities in Mukono District, Uganda. The outcome, Intention to disclose medical errors to patients, was defined as self-reported likelihood of informing a patient or their caregiver about a medical error if it had occurred, including the circumstances of error occurrence and any corrective actions taken. Data were collected using a self-administered structured questionnaire capturing factors at the individual and institutional levels. Logistic regression analysis was used to identify factors independently associated with intention to disclose medical errors, with adjusted odds ratios (aOR) and 95% confidence intervals (CI) reported. We studied 166 healthcare providers, of whom 90 (54.2%) were aged ≥35 years and 95 (57.2%) were female. Overall, 74 (44.6%) participants reported an intention to disclose medical errors. After adjusting for potential confounders, nurses (aOR 0.09, 95% CI 0.02-0.37) and other cadres (aOR 0.09, 95% CI 0.02-0.47) had significantly lower odds of intention to disclose medical errors compared with medical doctors/physicians. In contrast, healthcare providers working in health facilities with supportive supervision had higher odds of intention to disclose medical errors than those without supportive supervision (aOR 3.32, 95% CI 1.37-8.06). Fewer than half of healthcare providers have the intention to disclose medical errors to patients, indicating a substantial gap from the ethical expectation of full transparency. Supportive supervision was associated with an increased likelihood of intention to disclose medical errors, while non-physician cadres were less likely to report intention to disclose medical errors compared to medical doctors. Interventions that strengthen supervision and empower all cadres through training and institutional support may enhance openness and accountability in healthcare practice.

## Introduction

Medical errors remain a major global public health concern, contributing significantly to morbidity, mortality, and preventable healthcare costs. According to the World Health Organization [1], millions of deaths and disabilities occur annually due to medical errors, making them one of the leading causes of injury and mortality worldwide. In high-income countries such as the United States of America, estimates suggest that between 44,000 and 98,000 people die annually as a result of medical errors [2], while more recent evidence indicates that more than 250,000 deaths are attributable to such errors every year [3]. Similar findings have been reported in other settings, including Norway, where more than half of identified medical errors were harmful and nearly one percent fatal [4]. In low- and middle-income countries, including those in sub-Saharan Africa (SSA), medical errors are often underreported, and their impact on patient safety remains inadequately addressed, reflecting the urgent need for improved transparency and accountability in healthcare delivery.

Disclosure of medical errors to patients is a critical aspect of patient-centered care and an ethical obligation of healthcare providers [5]. Effective disclosure of medical errors promotes trust, strengthens patient–healthcare provider relationships, enhances communication, and contributes to organizational learning and patient safety [6]. However, global evidence indicates that healthcare providers often fail to disclose medical errors to patients. Studies have shown that less than one-third of medical errors are disclosed to affected patients or their families [7,8]. Factors such as fear of punishment, lack of institutional support, cultural stigma, inadequate reporting systems, and limited awareness of patients’ rights have been cited as barriers to disclosure of medical errors [9]. Within SSA, disclosure of medical error rates remains low, with previous studies in Nigeria and Ghana reporting disclosure rates of only 21.6% and near zero percent, respectively [10,11]. These low error reporting rates demonstrate a systemic gap in patient safety practices.

In Uganda, available evidence suggests that the problem of non-disclosure of medical errors persists, despite growing recognition of patient rights and safety. Although studies have explored the prevalence of medical errors in the past [12–14], few have examined factors influencing the intention to disclose medical errors to patients by healthcare providers. Data from a few Ugandan health facilities indicate that only 15–20% of medical errors are disclosed to supervisors or peers but rarely to patients [12,15]. The low medical error disclosure rates highlight the persistent challenges, such as fear of retribution, weak institutional frameworks, and the absence of standard operating procedures for disclosure.

There is a paucity of data on healthcare providers’ intention to disclose medical errors, an important precursor for medical error disclosure in practice, across Ugandan health facilities. The present study aimed to investigate the factors associated with intention to disclose medical errors by healthcare providers to patients at selected public health facilities in Uganda. Understanding these factors is essential to inform the design of effective interventions that promote transparency, strengthen patient safety culture, and support ethical and accountable healthcare practice in Uganda.

## Methods and materials

### Ethics statement

Ethical approval was obtained from the Bugema University Research Ethics Committee (BU-REC) under reference number BU-REC-2024–61. Administrative clearance was granted by the Mukono District Health Officer. Written informed consent was obtained from all participants before data collection. To protect confidentiality, no identifying information, such as names or staff numbers, was recorded. Participants were assured that participation was voluntary and that they could withdraw at any stage without consequences. Ethical Principles for research involving Human Subjects were adhered to as the study complied with the principles enunciated in the Declaration of Helsinki. Completed questionnaires were stored securely, and data were password-protected and accessible only to the research team.

### Study design and setting

This study employed an analytic cross-sectional design. This design is appropriate because it allows simultaneous assessment of exposures and outcomes at a point in time, providing a snapshot of prevailing practices and determinants of intention to disclose medical errors. The study was conducted across four public health facilities in Mukono District. The health facilities included the Mukono General Hospital, Kojja Health Centre IV, Nakisunga Health Centre III, and Kasawo Health Centre III. These facilities were purposively selected with guidance from the District Health Officer because they handle large patient volumes, offer a range of medical and surgical services, and represent different levels of care within the Ugandan health system. Reporting of the findings adhered to the Strengthening the Reporting of Observational studies in Epidemiology (STROBE) guidelines [16].

### Study population and eligibility criteria

The study population comprised healthcare providers directly involved in patient care, including medical officers, clinical officers, nurses, midwives, and laboratory professionals. Participants were eligible if they had worked at their health facility for at least one month and provided written informed consent. This ensured that the healthcare providers were familiar with the facility’s operations, including the occurrence, disclosure, and reporting of medical errors, to allow informed and accurate responses. Those who had served for less than one month or declined participation were excluded.

### Sampling and sample size determination

A total of 269 healthcare providers were available across the four selected health facilities at the time of the data collection. The sample size was determined using Yamane’s formula for finite populations, assuming a 5% margin of error. The calculated sample size was 160 participants, which was adjusted to 166 to account for a 3.5% non-response rate. Proportionate sampling was used to allocate the sample size across the four facilities, and participants were selected using simple random sampling from staff lists provided by the health facility heads.

Random numbers were generated in Stata version 15 and assigned to each staff member, and selection was conducted without replacement until the required number of participants per facility was achieved (Table 1).

**Table 1:** Sample size distribution across four study sites by proportionate to size.

Study sites	Number of healthcare providers	Proportion of healthcare providers	Sample size proportionate to size
A	39	0.15	25
B	38	0.14	24
C	56	0.21	35
D	136	0.51	85
**Total**	**269**	**1.00**	**166**

### Data collection methods

Data were collected between September 02, 2024, and December 13, 2024, using a structured, self-administered questionnaire written in English (S1 Data). The tool comprised three sections capturing healthcare worker characteristics, institutional factors, and disclosure practices. Questionnaires were distributed during staff meetings after explaining the study objectives, rationale, participant selection, and categories of medical errors, including preventive, diagnostic, treatment/procedural, and other errors such as communication, patient identification, and documentation errors. Thereafter, the participants completed the questionnaires privately, taking approximately 15–25 minutes. Data collection at each site lasted 2–3 days and was supported by two trained research assistants supervised by the principal investigator (AC). The research assistants received training on research ethics, study procedures, and data quality assurance to ensure uniformity and accuracy in data collection.

### Study variables and measurements

The main outcome was intention to disclose medical errors to patients. This was defined as the self-reported likelihood of informing a patient or their caregiver about a medical error if it had occurred, including the circumstances of error occurrence and any corrective actions taken. It was measured as a binary variable (yes = 1 vs. no = 0).

The individual-level independent variables included age, sex, professional cadreship, type of employment, years of experience, perceived workload, working hours, timing of error occurrence, and knowledge of medication errors. Age was measured as a continuous variable and further categorized into two groups: < 35 years and ≥35 years. Sex was recorded as male or female. Professional cadreship referred to the professional category of the healthcare worker and included medical doctors, clinical officers, nurses, midwives, and other related cadres. The type of employment described the contractual status of respondents and was classified as either permanent or temporary. Work experience was measured as the number of years in professional practice and categorized into ≤5 years and >5 years. Perceived workload captured respondents’ subjective assessment of their workload and was classified as either normal or heavy. Working hours were defined as the average number of hours worked per day and were dichotomized into ≤8 hours and >8 hours. The timing of error occurrence indicated the period during which medication errors most frequently occurred, and this was categorized as morning, afternoon, or night hours. Knowledge of disclosure of medical errors was categorized as either low or high.

The institutional-level independent variables included the level of the health facility, the existence of standard operating procedures, supervision, regular audits of medication errors, the existence of an error reporting system, and training on disclosure of medical errors. The level of health facility referred to the type of healthcare institution where respondents worked and was categorized as Health Center III, Health Center IV, or a hospital. The existence of standard operating procedures assessed whether the health facility had established and accessible written guidelines to standardize the dispensing process and prevent medication errors, classified as either “yes” or “no.” Supervision referred to the presence of regular oversight or monitoring of dispensing practices by senior staff or management, and was categorized as “yes” or “no.” Regular audits of medical errors measured whether the health facility conducted periodic reviews or evaluations to identify and correct errors, coded as “yes” or “no.” The existence of an error reporting system was assessed, whether a formal mechanism was available for reporting, documenting, and addressing medical errors within the health facility, also categorized as “yes” or “no.”

Lastly, training on disclosure of medical errors captured whether respondents or staff at the health facility had received any form of education or capacity-building (training) sessions related to medical errors, and was similarly categorized as “yes” or “no.”

### Data processing and statistical analysis

Data were checked for completeness, cleaned, and entered into EpiData version 3.1, integrated with quality control measures such as skips, alerts, and range and legal values to ensure data entry accuracy. Analysis was performed using R software version 4.2.1 (2022-06-23 ucrt, R Foundation for Statistical Computing, Vienna, Austria). Descriptive analysis comprised summarizing the participant characteristics and the outcome (intention to disclose medical errors) using frequency and percentages. Differences in the outcome across independent variables were evaluated using the Chi-square test for tables with all expected cell counts ≥5, and Fisher’s exact test when this assumption was not met. Variables with a p-value <0.05 in the bivariate analyses, those identified as relevant in the literature, or conceptually related to the outcome, were included in a multivariable logistic regression model to determine independent predictors of the outcome. Unadjusted/crude and adjusted odds ratios (aORs) with 95% confidence intervals (CIs) were reported to estimate the strength and direction of associations. Multicollinearity was assessed using the variance inflation factor (VIF), and variables with VIF ≥ 5 were excluded from the final regression model. We assessed the performance and adequacy of the logistic regression model using several complementary diagnostic approaches. Model discrimination was evaluated using the area under the receiver operating characteristic curve (AUC). Calibration was assessed using the Hosmer–Lemeshow goodness-of-fit test. Model specification was examined using the linktest, where a statistically significant linear predictor (_hat) and a non-significant squared predictor (_hatsq) were taken as evidence against major model misspecification. Model parsimony and relative fit were assessed using the Akaike Information Criterion (AIC), with lower values indicating better fit. Likelihood-ratio tests were used to compare nested models, with non-significant results indicating that additional model complexity did not significantly improve model fit.

### Inclusivity in global research

Information regarding ethical, cultural, and scientific considerations specific to inclusivity in global research is included in the Supporting Information (S1 Checklist).

## Results

### Distribution of participant characteristics by intention to disclose medical errors in Mukono District, Uganda

Of the 166 healthcare providers studied, 74 (44.6%) reported an intention to disclose medical errors to patients (Table 2). Differences in the intention to disclose medical errors were observed across several individual and institutional factors. Intention to disclose medical errors was higher among providers aged ≥35 years (54.1%), males (53.5%), medical doctors (83.3%), those with permanent employment (46.2%), more than five years of work experience (48.2%), those perceiving a normal workload (50.5%), and those working more than eight hours per day (47.8%). Systematic differences in intention to disclose medical errors were observed with respect to sex, professional cadre, availability of standard operating procedures, supervision, and training on disclosure of medical errors (all p < 0.05).

**Table 2:** Distribution of participant characteristics by intention to disclose medical errors in Mukono District, Uganda.

Variable	Level	Intention to disclose medical errors		p-value
		No, n (%)	Yes, n (%)	
Age categories (years)	<35	42 (45.7)	34 (45.9)	0.970
	≥35	50 (54.3)	40 (54.1)	
Sex	Male	33 (46.5)	38 (53.5)	0.045
	Female	59 (62.1)	36 (37.9)	
Professional cadreship	Medical Doctor	4 (16.7)	20 (83.3)	<0.001
	Clinical Officer	17 (44.7)	21 (55.3)	
	Nurse	45 (79.0)	12 (21.0)	
	Midwife	11 (39.3)	17 (60.7)	
	^+^Others	15 (79.0)	4 (21.0)	
Type of employment	Permanent	50 (53.8)	43 (46.2)	0.628
	Temporary	42 (57.5)	31 (42.5)	
Work experience in years	≤5	50 (58.8)	35 (41.2)	0.366
	>5	42 (51.9)	39 (48.2)	
Perceived workload	Normal	46 (49.5)	47 (50.5)	0.081
	Heavy			
Working hours	≤8	56 (57.7)	41 (42.3)	0.478
	>8	36 (52.2)	33 (47.8)	
Timing of medical error occurrence	Morning	20 (54.1)	17 (45.9)	0.940
	Afternoon	36 (54.6)	30 (45.4)	
	Night	36 (57.1)	27 (42.9)	
Knowledge of disclosure of medical errors	Low	41 (50.0)	41 (50.0)	0.165
	High	51 (60.7)	33 (39.3)	
Existence of standard operating procedures	No	65 (64.4)	36 (35.6)	0.004
	Yes	27 (41.5)	38 (58.5)	
Supportive supervision	No	38 (76.0)	12 (24.0)	<0.001
	Yes	54 (46.6)	62 (53.5)	
Regular audits of errors	No	65 (53.7)	56 (46.3)	0.469
	Yes	27 (60.0)	18 (40.0)	
Existence of an error reporting system	No	5 (33.3)	10 (66.7)	0.071
	Yes	87 (57.6)	64.42.4)	
Training on disclosure of medical errors	No	62 (66.0)	32 (34.0)	0.002
	Yes	30 (41.7)	42 (58.3)	

Note: + Refers to professionals such as laboratory, radiographers, pharmacy technicians, and pharmacists.

### Factors associated with intention to disclose medical errors by healthcare providers in Mukono District, Uganda

In the unadjusted analysis (Table 3), professional cadreship, the existence of standard operating procedures, and supervision were significantly associated with intention to disclose medical errors, while age, sex, and training on disclosure of medical errors were not. Specifically, nurses (Crude odds ratio [cOR] 0.05, 95% CI 0.02-0.19) and other healthcare cadres (cOR 0.05, 95% CI 0.01-0.25) were less likely to report intention to disclose medical errors to patients compared to medical doctors.

**Table 3:** Factors associated with intention to disclose medical errors by healthcare providers in Mukono District, Uganda.

Variable	Level	cOR (95% CI)	aOR (95% CI)
Sex	Male	1	1
	Female	0.53* (0.28–0.99)	0.81 (0.33–2.01)
Professional cadreship	Medical Doctor	1	1
	Clinical Officer	0.25* (0.07–0.86)	**0.36 (0.10–1.33)**
	Nurse	0.05*** (0.02–0.19)	0.09** (0.02–0.37)
	Midwife	0.31 (0.08–1.15)	0.65 (0.14–2.97)
	^+^Others	0.05*** (0.01–0.25)	0.09** (0.02–0.47)
Existence of standard operating procedures	No	1	1
	Yes	2.54** (1.34–4.82)	1.23 (0.45–3.34)
Supportive supervision	No	1	1
	Yes	3.64** (1.73–7.66)	3.32** (1.37–8.06)
Training on disclosure of medical errors	No	1	1
	Yes	2.71** (1.44–5.11)	1.36 (0.50–3.69)

**Note**: Statistical significance codes at 5% level: *P < 0.05, **P < 0.01, ***P < 0.001; Bolded figures indicate significant results. aOR: Adjusted odds ratio; cOR: Crude odds ratio; Note: + Refers to professionals such as laboratory, radiographers, pharmacy technicians, and pharmacists.

Healthcare providers in facilities with supervision (cOR 3.64, 95% CI 1.73-7.66) and those with standard operating procedures (cOR 2.54, 95% CI 1.34-4.82) had higher odds of intention to disclose medical errors.

After adjusting for age, sex, professional cadreship, existence of standard operating procedures, supervision, and training on disclosure of medical errors (Table 3), only professional cadreship and supervision remained independently associated with intention to disclose medical errors. Nurses (adjusted odds ratio [aOR] 0.09, 95% CI 0.02-0.37) and other cadres such as laboratory personnel (aOR 0.09, 95% CI 0.02-0.47) were less likely to report an intention to disclose medical errors. Healthcare providers working in health facilities with supervision were more likely to report intention to disclose medical errors (aOR 3.32, 95% CI 1.37-8.06). Age, sex, the existence of standard operating procedures, and training on disclosure of medical errors were not significantly associated with intention to disclose medical errors in the adjusted model.

### Model diagnostic results

Model diagnostics indicated acceptable performance. The model showed fair discrimination (AUC = 0.70; 95% CI: 0.62–0.78) and good calibration (Hosmer–Lemeshow χ² = 7.36, p = 0.498). The linktest showed no evidence of model misspecification (_hatsq p = 0.25). Model selection was supported by the lowest AIC, and likelihood-ratio testing indicated that more complex nested models did not significantly improve fit (p = 0.43). None of the variables had VIF ≥ 5.

## Discussion

This study investigated the factors associated with the intention to disclose medical errors by healthcare providers to patients in selected public health facilities in Mukono District, Uganda. This study found that nearly 45% of healthcare providers reported an intention to disclose medical errors to patients. This proportion is lower than the 64% medical error disclosure reported in Kenya [17], although direct comparison is limited by differences in outcome measurement, as the Kenyan study assessed actual disclosure practices rather than intention. Similarly, the proportion observed in this study is somewhat higher than the 35.4% prevalence of medical error reporting documented in two hospitals in Uganda [13], which focused on internal reporting rather than disclosure to patients. Taken together, and acknowledging these methodological differences, the findings suggest that both the intention to disclose and the practice of reporting or disclosing medical errors remain relatively limited in comparable East African healthcare settings. The relatively low intention to disclose medical error may be attributed to systemic, institutional, and personal barriers such as fear of blame, punitive or legal repercussions, inadequate supervision, and lack of formal training in error disclosure. These challenges are particularly common in resource-limited settings and reflect a culture of blame rather than learning, which discourages transparency and accountability when errors occur. A qualitative study that explored nurses’ attitudes and experiences regarding error disclosure, as well as perceived barriers, found that although most nurses believed patients should be informed about every error, only a few actually disclosed errors in practice [18].

We found that supportive supervision was associated with a higher likelihood of intention to disclose medical errors, although the association was attenuated as the confidence interval was wide. This finding aligns with an integrative review that reported supportive environment, clear supervisory processes, and leadership encouragement enhance nurses’ willingness to report and disclose errors [19]. It is also consistent with the findings of a systematic review that reported regular, trusting, and private supervision, along with leadership support, are enablers of open communication—a prerequisite for intention to disclose error or error disclosure in practice [20]. Similarly, systematic review evidence across multiple settings has shown that organizational culture and supervisory support are consistent enablers of error disclosure and reporting [21]. Furthermore, another study found that closer and structured supervision reduces medical errors and improves safety behaviors, with follow-on studies interpreting this to include improved error management and disclosure practices when supervisors actively support trainees [22]. In contrast, Zaghloul and colleagues [23] found no significant relationship between supervision and medical error disclosure, indicating that supervision quality and context may determine its effectiveness. The present study supports the argument that effective supervision creates an enabling environment through mentorship, accountability, and reassurance against punitive consequences, hence may improve the intention to disclose medical errors or medical error disclosure in practice. Such a culture not only facilitates learning from mistakes but also strengthens trust between healthcare providers and patients.

Furthermore, intention to disclose medical errors is less likely among non-physician cadres, namely clinical officers, nurses, and midwives, compared to medical doctors, although the finding is imprecise. In Uganda’s hierarchical healthcare system and across many countries in SSA, doctors often receive more extensive training in medical ethics, patient communication, and professional autonomy, which may empower them to disclose errors more confidently. In contrast, non-physician cadres often lack such empowerment and face greater pressure from supervisors or institutional policies that discourage transparency. Our finding is supported by various studies that all reported that lower-cadre healthcare providers tend to underreport or conceal medical errors due to fear of disciplinary action, lack of authority, and inadequate ethical training [24,25]. However, one study found no significant association between professional cadre and disclosure of medical errors [26], suggesting that contextual and institutional factors may modify this relationship.

### Implications of findings for practice, research, and policy

The findings underscore the urgent need to institutionalize non-punitive approaches to medical error management and disclosure. Health facilities should strengthen supportive supervision systems that promote mentorship, learning, and psychological safety. In addition, targeted training programs on patient communication, ethics, and medical error disclosure protocols should be extended to all cadres of healthcare providers, particularly non-physicians. At a policy level, integrating medical error disclosure guidelines into Uganda’s national patient safety framework would help standardize practices and promote a culture of openness and accountability across all health facilities.

### Study strengths and limitations

This study provides valuable insights into the intention to disclose medical errors among healthcare providers in Uganda and other low-resource settings, using a representative sample across four health facilities and a rigorous quantitative approach. The use of structured questionnaires allowed for standardized data collection and reliable assessment of both individual and institutional factors influencing the intention to disclose medical errors. However, there are limitations to consider in the interpretation of the findings. The cross-sectional design limits causal inference, and self-reported data may be subject to social desirability bias, potentially underestimating or overestimating disclosure practices. Also, the study was conducted in selected public health facilities in Mukono District, which may limit generalizability to private healthcare settings. Our outcome was relatively common; therefore, the prevalence odds ratio may have overestimated the strength of the association compared with the prevalence risk ratio. While penalized regression approaches could further address small-sample bias, we did not apply these methods and acknowledge this as a limitation. Cadre type was included as a covariate to partially account for role-related differences, although residual confounding due to unmeasured patient-contact intensity may remain.

The purposive sampling of health facilities may limit the generalizability of the findings, as facilities not included in the study may have yielded different data. Lastly, our sample size was relatively small, and this resulted in wide confidence intervals or imprecise findings. Despite these limitations, the findings offer actionable evidence to inform interventions aimed at improving error disclosure and patient safety in Uganda and similar settings in SSA.

## Conclusion and recommendations

Fewer than half of healthcare providers intend to disclose medical errors to patients, indicating a substantial gap from the ethical expectation of full transparency. Supportive supervision was associated with an increased likelihood of intention to disclose medical errors, while non-physician cadres were less likely to intend to disclose medical errors compared to medical doctors. Interventions that strengthen supervision and empower all cadres through training and institutional support may enhance openness and accountability in healthcare practice.

## Supporting information

S1 Data Dataset. (CSV)

S1 Checklist Inclusivity in global health research. (DOCX)

## References

[1] World Health Organization (WHO). Patient safety. Geneva, Switzerland: WHO; 2025 [cited 2025 May 5]. Available from: https://www.who.int/news-room/fact-sheets/detail/patient-safety

[2] Institute of Medicine Committee on Quality of Health Care in America. To Err is Human: Building a Safer Health System. In: Kohn LT, Corrigan JM, Donaldson MS, editors. Washington (DC): National Academies Press (US); 2000. 25077248

[3] Makary MA, Daniel M. Medical error-the third leading cause of death in the US. BMJ. 2016;353:i2139. doi: 10.1136/bmj.i2139 27143499

[4] Mulac A. Medication errors in hospitals: Exploring medication safety through incident reports and observation of practice. 2022.

[5] Radwan MA. Disclosing adverse events in healthcare: understanding the gap between knowledge and practice in developing countries. Glob J Qual Saf Healthc. 2022;5(4):82–3. doi: 10.36401/JQSH-22-X4 37260934 PMC10229041

[6] Wu AW, McCay L, Levinson W, Iedema R, Wallace G, Boyle DJ, et al. Disclosing adverse events to patients: international norms and trends. J Patient Saf. 2017;13(1):43–9. doi: 10.1097/PTS.0000000000000107 24717530

[7] Gallagher TH, Waterman AD, Ebers AG, Fraser VJ, Levinson W. Patients’ and physicians’ attitudes regarding the disclosure of medical errors. JAMA. 2003;289(8):1001–7. doi: 10.1001/jama.289.8.1001 12597752

[8] Kaldjian LC, Jones EW, Rosenthal GE, Tripp-Reimer T, Hillis SL. An empirically derived taxonomy of factors affecting physicians’ willingness to disclose medical errors. J Gen Intern Med. 2006;21(9):942–8. doi: 10.1111/j.1525-1497.2006.00489.x 16918739 PMC1831592

[9] Al Rowily A, Jalal Z, Price MJ, Abutaleb MH, Almodiaemgh H, Al Ammari M, et al. Prevalence, contributory factors and severity of medication errors associated with direct-acting oral anticoagulants in adult patients: a systematic review and meta-analysis. Eur J Clin Pharmacol. 2022;78(4):623–45. doi: 10.1007/s00228-021-03212-y 34935068 PMC8926953

[10] Ogundiran TO, Adebamowo CA. Surgeon-patient information disclosure practices in southwestern Nigeria. Med Princ Pract. 2012;21(3):238–43. doi: 10.1159/000333817 22123339 PMC6902227

[11] Alhassan RK, Halilu B, Benin SM, Donyor BF, Kuwaru AY, Yipaalanaa D, et al. Experiences of frontline nurses with adverse medical events in a regional referral hospital in northern Ghana: a cross-sectional study. Trop Med Health. 2019;47:36. doi: 10.1186/s41182-019-0163-8 31160884 PMC6537203

[12] Katongole SP, Anguyo RD, Nanyingi M, Nakiwala SR. Common medical errors and error reporting systems in selected hospitals of central Uganda. 2015.

[13] Mauti G, Githae M. Medical error reporting among physicians and nurses in Uganda. Afr Health Sci. 2019;19(4):3107–17. doi: 10.4314/ahs.v19i4.33 32127887 PMC7040326

[14] Dorothy A, Yadesa TM, Atukunda E. Prevalence of medication errors and the associated factors: a prospective observational study among cancer patients at Mbarara Regional Referral Hospital. Cancer Manag Res. 2021;13:3739–48. doi: 10.2147/CMAR.S307001 34007209 PMC8121619

[15] Kavuma M, Mars M. The effect of an integrated electronic medical record system on malaria out-patient case management in a Ugandan health facility. Health Informatics J. 2022;28(4). doi: 10.1177/14604582221137446 36345921

[16] von Elm E, Altman DG, Egger M, Pocock SJ, Gøtzsche PC, Vandenbroucke JP, et al. Strengthening the Reporting of Observational Studies in Epidemiology (STROBE) statement: guidelines for reporting observational studies. BMJ. 2007;335(7624):806–8. doi: 10.1136/bmj.39335.541782.AD 17947786 PMC2034723

[17] Ngivu JN. Factors influencing reporting of medical errors amongst nurses in pediatric wards in three teaching and referral hospitals in Nairobi Kenya. KeMU; 2022.

[18] McLennan SR, Diebold M, Rich LE, Elger BS. Nurses’ perspectives regarding the disclosure of errors to patients: A qualitative study. Int J Nurs Stud. 2016;54:16–22. doi: 10.1016/j.ijnurstu.2014.10.001 25458803

[19] Woo MWJ, Avery MJ. Nurses’ experiences in voluntary error reporting: an integrative literature review. Int J Nurs Sci. 2021;8(4):453–69. doi: 10.1016/j.ijnss.2021.07.004 34631996 PMC8488811

[20] Rothwell C, Kehoe A, Farook SF, Illing J. Enablers and barriers to effective clinical supervision in the workplace: a rapid evidence review. BMJ Open. 2021;11(9):e052929. doi: 10.1136/bmjopen-2021-052929 34588261 PMC8479981

[21] Vaismoradi M, Vizcaya-Moreno F, Jordan S, Gåre Kymre I, Kangasniemi M. Disclosing and reporting practice errors by nurses in residential long-term care settings: a systematic review. Sustainability. 2020;12(7):2630. doi: 10.3390/su12072630

[22] Tamuz M, Giardina TD, Thomas EJ, Menon S, Singh H. Rethinking resident supervision to improve safety: from hierarchical to interprofessional models. J Hosp Med. 2011;6(8):445–52. doi: 10.1002/jhm.919 21990173 PMC3201712

[23] Zaghloul AA, Rahman SA, Abou El-Enein NY. Obligation towards medical errors disclosure at a tertiary care hospital in Dubai, UAE. Int J Risk Saf Med. 2016;28(2):93–9. doi: 10.3233/jrs-160722 27567766 PMC5008227

[24] Abdalla EA, Abdoon IH, Osman B, Osman WJA, Mohamed EM. Perception of medication errors’ causes and reporting among Sudanese nurses in teaching hospitals. Appl Nurs Res. 2020;51:151207. doi: 10.1016/j.apnr.2019.151207 31676299

[25] Hs A-S, Rashid A. The intention to disclose medical errors among doctors in a referral hospital in North Malaysia. BMC Med Ethics. 2017;18(1):3. doi: 10.1186/s12910-016-0161-x 28114911 PMC5259943

[26] Sabblah GT, van Hunsel F, Taxis K, Duwiejua M, Seaneke SK, van Puijenbroek E. Medication errors by caregivers in the homes of children discharged from a pediatric department in Ghana. Ther Adv Drug Saf. 2024;15. doi: 10.1177/20420986231225850 38293565 PMC10823839

